# Disease of the Nose and Throat

**Published:** 1900-05

**Authors:** 


### BOOK REVIEWS, PAMPHLETS, EXCHANGES.

### BOOK REVIEWS.

[Contributions solicited for review.]

DISEASE OF THE NOSE AND THROAT. By J. Price-Brown, M.B., L.R.C.P.E., Member of the College of Physicians and Surgeons of Ontario; Laryngologist to the Toronto Western Hospital; Laryngologist to the Protestant Orphans' Home; Fellow of the American Laryngological, Rhinological, and Otological Society; Member of the British Medical Association, the Pan-American Medical Congress, the Canadian Medical Association, the Ontario Medical Association, etc., etc. Illustrated with 159 Engravings, including 6 Full-Page Color-plates and 9 Color-cuts in the text, many of them Original. 6¼ x 9¼ inches. Pages xvi–470. Extra Cloth, $3.50 net. The F. A. Davis Co., Publishers, 1914-16 Cherry St., Philadelphia.

It is difficult to see why any author should produce a small manual or text-book on diseases of the nose and throat when there are so many of the same nature now before the profession and so many of them produced within the last two years. Dr. Brown has produced a neat little book, but throughout its pages there is nothing original. The discussion of the various subjects reminds one more of a "compend" than of a text-book. The author gives due credit to those from whom he has borrowed, which is indeed commendable. The illustrations are almost entirely from the works of Bosworth and Lennox Browne, and as such are splendid. It may be that the author expects his book to fill a place needed among the Canadian profession, and we trust that such may be the case. R.

THE ATLAS OF LEGAL MEDICINE. By Dr. E. Von Hoffman, Professor of Legal Medicine and Director of the Medico-Legal Institute at Vienna.

This is one of the incomparable hand-atlas series published by W. B. Saunders of Philadelphia. We owe the publishers an apology for overlooking it in our reviews, for no one of this notable series has been more highly prized or oftener consulted in this office. It is translated by Professor A. O. J. Kelly of the University of Pennsylvania. There are 196 illustrations and 56 full-sized plates, with as many pages of explanatory text. This book will prove of the greatest value to coroners and lawyers in the study of criminal cases. The price is $3.50 net. Without the foresight and liberality of Mr. Saunders this book would only be accessible in the German tongue and at a much higher price. The plates and text relating to criminal abortion alone are well worth the price of the book.

---

PRACTICAL ANATOMY, INCLUDING A SPECIAL SECTION ON THE FUNDAMENTAL PRINCIPLES OF ANATOMY. Edited by W. T. Eckley, M.D., Professor of Anatomy in the College of Physicians and Surgeons, University of Illinois; Professor of Anatomy in the Northwestern University Dental School; Professor of Anatomy in the Chicago Clinical School, and Director of the Chicago School of Anatomy and Physiology; Member of the American Medical Association, the Chicago Pathological Society, the Chicago Medical Society, etc.; and Mrs. Corinne Buford Eckley, Instructor of Anatomy in the Northwestern University Dental School; Professor of Anatomy in the Northwestern University Woman's Medical School; Professor of Anatomy in the Chicago School of Anatomy and Physiology. With 347 illustrations, many of which are in colors. Octavo. Price $3.50 net, Cloth; $4.00 net, Oil Cloth. P. Blakiston's Sons & Co., 1012 Walnut St., Philadelphia.

This book is intended as a dissecting-room guide to Morris's Anatomy. It is very clear and explicit, and will be of great use to the student in practical work in anatomy. The illustrations are numerous and accurately executed. It admirably fulfils its purpose.

